# Relationship between structural order and water-like anomalies in metastable liquid silicon: *Ab initio* molecular dynamics

**DOI:** 10.1038/srep39952

**Published:** 2017-01-05

**Authors:** G. Zhao, J. L. Yan, Y. J. Yu, M. C. Ding, X. G. Zhao, H. Y. Wang

**Affiliations:** 1 School of Physics and Optoelectronic Engineering, Ludong University, Yantai 264025, P. R. China

## Abstract

The relationship between structural order and water-like anomalies in tetrahedral liquids is still open. Here, first-principle molecular dynamics are performed to study it in metastable liquid Si. It is found that in *T*-*P* phase diagram, there indeed exists a structural anomaly region, which encloses density anomaly but not diffusivity anomaly. This is consistent with that of SW Si and BKS SiO_2_ but different from that of SPC/E water. Two-body excess entropy anomaly can neither capture the diffusivity, structural, and density anomalies, as it can in a two-scale potential fluid. In structural anomaly region, tetrahedrality order *q*_*tetra*_ (measuring the extent to which an atom and its four nearest neighbours adopt tetrahedral arrangement) and translational order *t*_*trans*_ (measuring the tendency of two atoms to adopt preferential separation) are not perfectly correlated, which is different from that in SW Si and renders it impossible to use the isotaxis line to quantify the degree of structural order needed for water-like anomalies to occur. Along the isotherm of critical temperature *T*_*c*_, *t*_*trans*_/*q*_*tetra*_ is approximately linear with pressure. With decreasing pressure along the isotherm below *T*_*c*_, *t*_*trans*_/*q*_*tetra*_ departs downward from the line, while it is the opposite case above *T*_*c*_.

Tetrahedral liquids, in which the anisotopic bonding gives rise to a three-dimensional, liquid-state network with an energetic bias towards local tetrahedral order, are ubiquitous in everyday life, such as water, SiO_2_, BeF_2_, GeO_2_, C, Si, Ge, Sn. Although the interactions between atoms or molecules are different, including ionic, metallic, covalent and hydrogen bonding, they share a preference for forming tetrahedrally coordinated configurations at the micro level and displaying water-like anomalous behaviour at the macro level, such as the well-known density maximum (density anomaly)^1^, the anomalous increase of diffusivity upon pressurizing (diffusivity anomaly)^2^,^3^,^4^,^5^,^6^, and the first-order liquid-liquid phase transition (LLPT)^7^,^8^,^9^,^10^,^11^,^12^,^13^,^14^,^15^,^16^,^17^,^18^. Tetrahedral liquids form a large fraction of our world and have fundamental biological and technological relevance. Therefore, a general interpretation of these anomalous behaviours is of wide interest.

In recent years, much effort has been expended to understand the relationship between the atomic level structure and the water-like anomalies. In 2001, Errington and Debenedetti^6^ firstly introduced two measures of order, the translational order parameter (*t*_*trans*_)^19^ and the tetrahedrality order parameter (*q*_*tetra*_)^20^, to analyse the local structural order in the SPC/E model of water and further to study the relationship between structural order and these anomalies. It was found that, in the region bounded by loci of maximum *q*_*tetra*_ at low densities and minimum *t*_*trans*_ at high densities along the isotherm, *q*_*tetra*_ and *t*_*trans*_ decrease on compression, in contrast to what is observed for simple fluids. Therefore, this region was identified by the authors as a structurally anomalous region, which in turn encloses the regions of diffusivity anomaly and density anomaly in the *T*-*ρ* phase diagram. Moreover, the two orders were found to be strictly coupled in this region, i.e., a particular value of *q*_*tetra*_ is associated with a unique value of *t*_*trans*_, and so the isotaxis lines (from the Greek word taxis, meaning order, or arrangement) can be used to quantify the degree of structural order needed for these anomalies to occur. Soon after that, using the Beest-Kramer-van Santen (BKS) model potential, a similar analysis in liquid silica was carried out by Shell *et al*.^21^. However, they found that even though there also exists a structurally anomalous region similar to that in water and it encloses the density anomaly, it does not encompass the region of diffusivity anomaly and the structural order parameters *q*_*tetra*_ and *t*_*trans*_ are not strictly coupled in this region. Therefore, they concluded that *q*_*tetra*_ and *t*_*trans*_ can not anticipate the occurrence of the diffusivity anomaly and hence can not provide a satisfactory microscopic picture of the anomalous behaviour in liquid silica. The later results of liquid BeF_2_ with the transferable rigid ion model (TRIM) by Agarwal *et al*.^22^ shows the similar result with BKS SiO_2_, but that of the Oeffner-Elliot (OE) model of GeO_2_ by Jabes *et al*.^23^ is similar to SPC/E water. The systematic comparison for different model potentials of water (mTIP3P, TIP4P, TIP4P/2005, TIP5P, SPC/E), ionic liquids (SiO_**2**_ and BeF_**2**_), and liquids characterised by the Stillinger-Weber (SW) potential (including Si)^24^,^25^,^26^,^27^ suggests that a strong correlation between *q*_*tetra*_ and *t*_*trans*_ exists only in rigid-body model potentials for water and SW liquids (in a limited range of tetrahedrality strength), but not in ionic melts. In addition, to obtain a more general picture of the origins of these anomalies, the excess entropy has also been attempted to understand these anomalies and has been tested to be able to predict the regions of anomalies in the phase diagram^22^,^23^,^24^,^25^,^26^,^27^,^28^,^29^.

It should be noted that the water-like cascading of anomalous regions in the phase diagram has been observed not only in systems having directional interactions but also in the simulation studies of spherically symmetric potentials^29^,^30^,^31^,^32^,^33^. Especially, the study of Jagla model by Yan *et al*.^30^ suggests the water-like relationship between structural order and anomalies is related to the presence of two different length scales in the potential. Moreover, the study of a core-softened model by Fomin *et al*.^33^ shows that the order of the cascading regions of anomaly can be changed by increasing the depth of the attractive part of the potential.

Although so many computer simulation studies of tetrahedral liquids have focused on the relationship between structural order and water-like anomalies, they all depended on different model potentials or empirical potentials. To our knowledge, first-principle computer simulations have not been performed to study this question so far. It is well-known that the first-principle force neither makes assumptions such as empirical model nor includes fitting parameter to experimental data, so it is expected to give more realistic results. In the past few decades, first-principle simulations had achieved great success in investigating structural and dynamic properties of liquids. Based on first-principle calculations, we have studied the inherent structures of high-density liquid (HDL) and low-density liquid (LDL) Si and found that the first-order LLPT in metastable liquid Si is a transition between a *sp*^3^-hybridization LDL and a white-tin-like HDL^34^. Further, we found that the liquid-liquid critical point (LLCP) is hidden, blended in with the continuous reentrant spinodal of HDL, suggesting that the phase behaviour of metastable liquid Si tends to be a critical-point-free scenario rather than a second-critical-point one based on SW potential^35^. In this work, based on the prior research, we turn our attention to the relationship between structural order and water-like anomalies (including the well-known first-order LLPT) in metastable liquid silicon.

## Results

The density anomaly and the first-order LLPT have been shown in our previous work^35^. In the studied pressure range from −27.25 to 6.50 kbar, an obvious maximum can be observed along each *ρ*-*T* isobar line^35^ and in *T*-*P* phase diagram, the line of density maxima tends to pass through the LLCP, located at *T*_*c*_ ~ 1420 ± 10 K and *P*_*c*_ ~ −30.5 ± 1.0 kbar^35^, which is different from the result in ref. 17 (*T*_*c*_ ~ 1120 K and *P*_*c*_ ~ 0.60 GPa). We speculate that such a difference maybe origins from the different potentials adopted. Although the LLPT is proved to exist for SW liquid Si, it has been reported that it strongly depends on the parameters of the potential and an arbitrary variation, albeit small, of the parameters may even lead to its disappearance^36^. Here, we begin by presenting our results related to the diffusivity anomaly.

### Diffusivity anomaly

**Figure 1:**
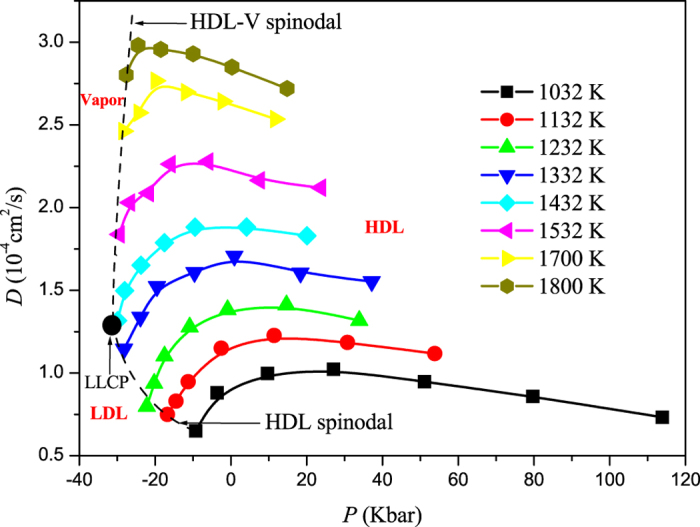
Diffusivity as a function of pressure for different temperatures from 1032 to 1800 K obtained from *ab initio* molecular dynamics simulations of liquid Si. The maximum in diffusivity demarcates the regions of normal and anomalous behaviour in *T*-*P* phase diagram.

In Fig. 1, we show our calculated diffusion coefficient *D* as a function of pressure along eight different isotherms ranging from 1032 to 1800 K. Along each isotherm, the pressure is gradually decreased to the stability limit of HDL and an obvious maximum can be found in the whole temperature range studied here, which marks the upper bound of diffusivity anomaly region. Along each isotherm below *T*_*c*_, the locus of diffusivity minimum, which marks the lower bound of diffusivity anomaly region, can not be found and is expected to be located in LDL, where system’s dynamics is slow and there is considerable error in computing *D* and locating the diffusivity minima. So we have not attempted to find this locus in our study. Above *T*_*c*_, the diffusivity minimum is expected to be located at the HDL-V spinodal because of the larger *D* in vapor than that in liquid.

### Structural anomaly

**Figure 2:**
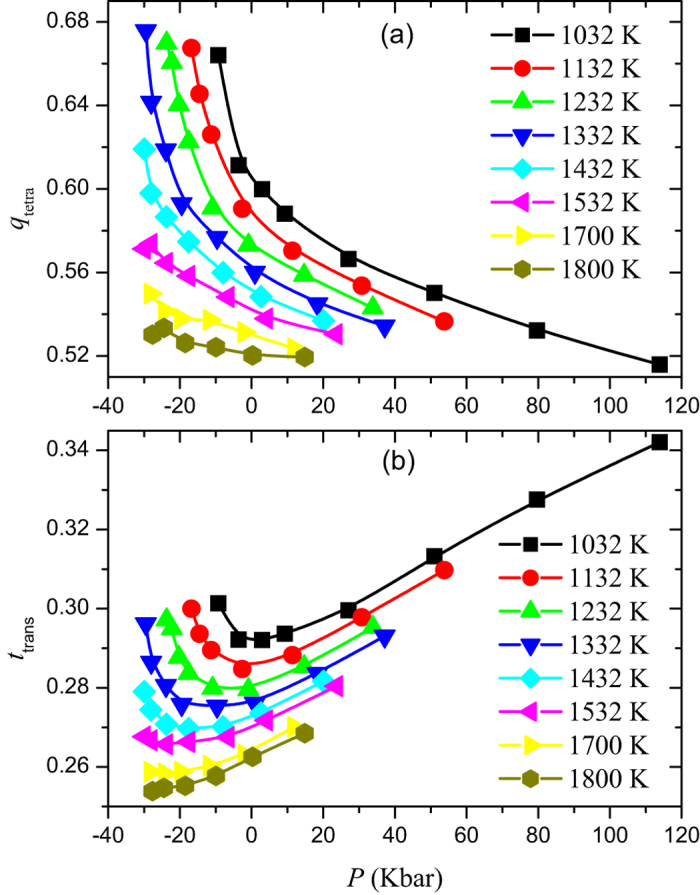
The average tetrahedrality order *q*_*tetra*_ (**a**) and translational order *t*_*trans*_ (**b**) as a function of pressure for different temperatures from 1032 to 1800 K obtained from *ab initio* molecular dynamics simulations of liquid Si. *q*_*tetra*_ increases with decreasing pressure. From 1032 to 1700 K, *t*_*trans*_ decreases with decreasing pressure and goes through a minimum, which marks an onset of structural anomaly.

Figure 2 presents the evolution of structural order (*t*_*trans*_ and *q*_*tetra*_) with decreasing pressure along the isotherm. From Fig. 2(a), it can be found that *q*_*tetra*_ increases with the decrease of pressure in the simulated temperature range from 1032 to 1800 K, which is in contrast to what is observed for simple fluids and so is termed as structural anomaly. Below *T*_*c*_, we do not find a maximum of *q*_*tetra*_ in our simulated pressure range, which marks the lower bound of structural anomaly region and is expected to be found in LDL. At temperatures above *T*_*c*_, the maximum of *q*_*tetra*_ is expected to be located at the HDL-V spinodal, such as for *T* = 1532 and 1800 K. In Fig. 2(b), in the temperature range from 1032 to 1700 K, *t*_*trans*_ decreases with decreasing pressure and goes through a minimum, which marks the upper bound of structural anomaly region as suggested by Errington and Debenedetti^6^. For temperature *T* = 1800 K, the minimum cannot be found until the HDL-V spinodal is encountered.

### Excess entropy anomaly

**Figure 3:**
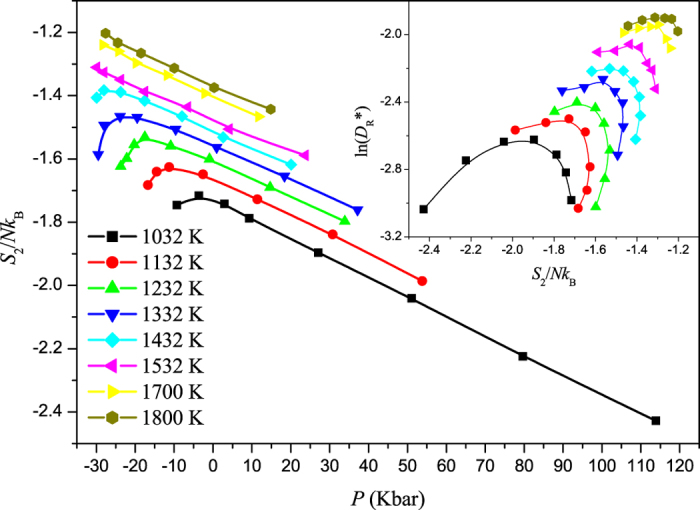
Two-body excess entropy *S*_2_ of liquid Si as function of pressure for different temperatures from 1032 to 1800 K obtained from the pair correlation function. *S*_2_ decreases anomalously with decreasing pressure after going through a maximum, for temperature ranging from 1032 to 1432 K. Inset: The Rosenfeld reduced diffusion coefficient D* against *S*_2_ for different isotherms. The relation between ln(*D*_R_*) and *S*_2_ is not linear, showing a different behaviour from that of Rosenfeld and Dzugutov.

The excess entropy *S*_*ex*_ is defined as the entropy of the liquid relative to that of the ideal gas at the same temperature and pressure and can be expressed in terms of an expansion of *n*-body correlation functions. The first term, namely the two-body contribution *S*_2_, accounts for a large proportion, such as 80–90% in Lennard-Jones fluids^37^. Because *S*_*ex*_ characterizes the reduction in the number of states (relative to an ideal gas) accessible to a system due to translational interparticle correlations, it can also be thought of as a metric for translational order. Errington and co-workers^29^ argued that the region in the phase diagram where the excess entropy anomalously increases with decrease in density is also the region where translational order anomaly is observed. Indeed, many studies mentioned above have showed that there also exists a *S*_*ex*_ or *S*_2_ anomaly region in the *T*-*ρ* phase diagram, where *S*_*ex*_ or *S*_2_ anomalously increases with increasing pressure. In this work, we calculated *S*_2_ as a function of pressure along eight different isotherms, shown in Fig. 3. In the temperature range from 1032 to 1432 K, along each isotherm a maximum occurs but the minimum can not be found until the HDL spinodal is encountered, which is expected to be found in LDL. From 1532 to 1800 K, the maximum can not be found until the HDL-V spinodal is encountered.

It is known that there is a strong connection between transport properties of fluids and excess entropy, such as the Rosenfeld scaling relation *D*_R_* = *a*_*D*_exp(*b*_*D*_*S*_*E*_)^38^,^39^ and the Dzugutov scaling relation *D*_z_* = *a*_0_exp(*S*_2_)^40^. In the former, the reduced diffusion coefficient *D*_R_* = *Dρ*^1/3^/(*k*_*B*_*T*/*m*)^1/2^, *m* is the mass of the particle, and *ρ* is the number density. In the later, *D*_z_* = *D*Γ^−1^*σ*^−2^, Γ is the collision frequency and equals 4*σ*^2^*g(σ)ρ(πk*_*B*_*T*/*m*)^1/2^, *σ* is the hard-sphere diameter, and g(*σ*) is the value of radial distribution function at the constant distance. In Fig. 3 (inset), we also test the scaling relation between *D*_R_* and *S*_2_. The relation between *D*_z_* and *S*_2_ is similar to that presented in Fig. 3 (inset) and not shown here. However, it can be seen that the relation between ln(*D*_R_*) and *S*_2_ is not linear, showing a different behaviour from that of Rosenfeld and Dzugutov. Based on the results in Figs 1 and 3, it is due to the fact that along an isotherm, the change of diffusivity with pressure is out of step with that of *S*_2_ versus pressure, which is different from the results in ref. 41 based on a core-softened potential (i.e., a Lennard-Jones potential plus a Gaussian repulsion). Thus we can comment that, based on first-principle calculations, the Rosenfeld and Dzugutov scaling relations are invalid along an isotherm in the anomalous region. This is consistent with that observed in supercooled liquid Si based on SW potential^27^ and the systems based on some two-scale potentials^42^,^43^.

## Discussion

**Figure 4:**
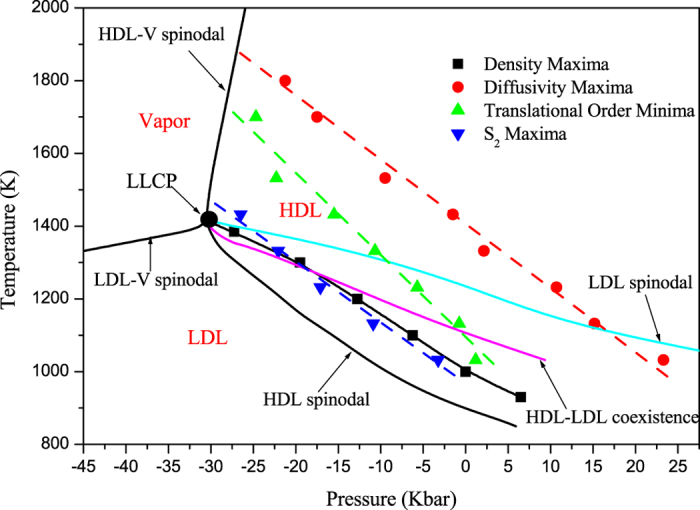
*T*-*P* phase diagram of liquid silicon showing the loci of density maxima, diffusivity maxima, translational order minima and two-body excess entropy maxima along other features of the phase diagram.

Based on the above results, now we turn to discuss the relationship of these anomalies in *T*-*P* phase diagram, shown in Fig. 4. The spinodals, LLCP, HDL-LDL coexistence line, and density maxima are obtained from our previous work^35^. The LDL-V and the HDL-V spinodals form a continuous liquid-vapor spinodal, which meets the liquid-vapor critical point at higher temperature and higher pressure (not shown here), and the LLCP seems to just be hidden, blended in with the liquid-vapour spinodal. This is consistent with the critical-point-free scenario suggested by Angell in refs 44 and 45, but is different with the two-critical-point scenario, in which the LLCP occurs above the liquid-vapor spinodal, similar to that presented in Fig. 2 in ref. 46. From Fig. 4, it can be seen that the region of diffusivity anomaly encloses the region of structural anomaly, which in turn encloses the region of density anomaly. This result is consistent with that of SW Si^27^ and is similar to that of BKS SiO_2_^21^ but different from that of SPC/E model of water^6^. In addition, it should be noticed that the two-body excess entropy anomaly region does not enclose the diffusivity anomaly as it does in a fluid interacting through a two-scale potential^29^. On the contrary, it is also included in the structural anomaly region determined by the translational order minima, with the *S*_2_ maxima line going across the density maxima line. So *S*_2_ anomaly can not be used as a criteria of structural anomaly to capture the region of diffusivity and density anomalies. This may origin in the fact that the relation between ln(*D*_R_*) and *S*_2_ shows an anomalous behaviour, presented in Fig. 3 (inset).

Recently, it has been shown for the two-scale potential that different criteria of structural anomaly can lead to completely different results^47^. It was found that the structural anomaly region determined by the *S*_*ex*_ anomaly lies much deeper than that obtained from the approximate *S*_2_ anomaly. This means that the structural anomaly calculated from the *S*_*ex*_ criterion may be inside the density anomaly shown in Fig. 4, which is in contradiction with the thermodynamically consistent relation that the region of anomalous density is always inside the region of anomalous *S*_*ex*_^47^. We think that there are two main reasons accountable for this. The first is that there is a larger error in determining the temperature of density maximum, the higher the pressure, the greater the error (see Fig. 3 in ref. 35). The second is that, although the structural anomaly region determined by *S*_*ex*_ lies much deeper than that obtained from *S*_2_ for the two-scale potential^47^, it is not necessarily the case for other systems. For example, in SW Si the onset pressure of *S*_*ex*_ anomaly is the same with that of *S*_2_ anomaly and at lower temperatures the former is even slightly larger than the latter (see Fig. 12(a) in ref. 27).

**Figure 5:**
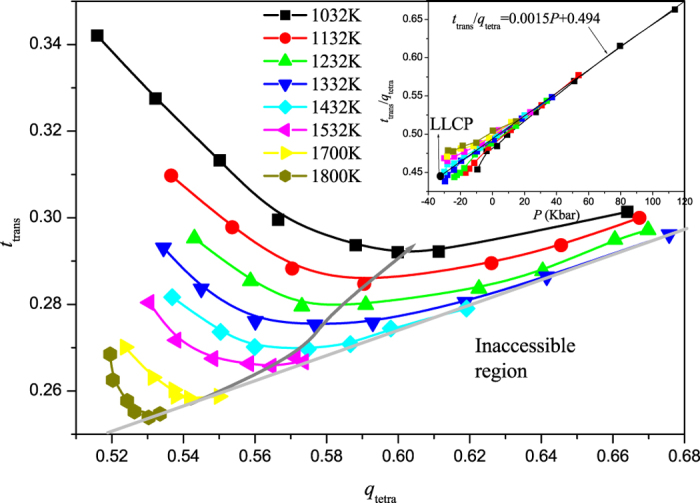
Parametric plot of translational order *t*_*trans*_ against tetrahedrality order *q*_*tetra*_ for different temperatures from 1032 to 1800 K obtained from *ab initio* molecular dynamics simulations of liquid Si. *q*_*tetra*_ and *t*_*trans*_ are not perfectly correlated in the structural anomaly region and with decreasing temperature, the correlation becomes weaker and weaker. Inset: The value of *t*_*trans*_/*q*_*tetra*_ against pressure for different isotherms. Along the isotherm of *T*_*c*_, *t*_*trans*_/*q*_*tetra*_ is approximately linear with pressure, *t*_*trans*_/*q*_*tetra*_ = 0.0015*P* + 0.494. With decreasing pressure along the isotherm below *T*_*c*_, *t*_*trans*_/*q*_*tetra*_ departs downward from the line until the LLPT occurs, while it is the opposite case above *T*_*c*_.

As mentioned above, in the structural anomaly region, a strong correlation between *q*_*tetra*_ and *t*_*trans*_ exists in rigid-body model potentials for water and SW liquids (in a limited range of tetrahedrality strength), but not in ionic melts. Here, we also present the correlation between *q*_*tetra*_ and *t*_*trans*_, shown in Fig. 5. Similar to water, our Si order parameter map also suggests an inaccessible region in the high-*q*_*tetra*_, low-*t*_*trans*_ quadrant. In other words, for any given value of *t*_*trans*_, there is a maximum value of *q*_*tetra*_ that the system can attain. Likewise, for any given value of *q*_*tetra*_, there is a minimum attainable *t*_*trans*_. However, in the structural anomaly region, *q*_*tetra*_ and *t*_*trans*_ do not collapse onto a single line, as is the case for water, suggesting that *q*_*tetra*_ and *t*_*trans*_ are not perfectly correlated. With the decrease of temperature, the correlation becomes weaker and weaker. These results are different from those observed in SW Si^25^,^26^,^27^, in which *q*_*tetra*_ and *t*_*trans*_ are perfectly correlated in the structural anomaly region. This degree of independence renders it impossible to use the isotaxis line to quantify the degree of structural order needed for the water-like anomalies to occur.

To obtain the relationship between structural order and the LLPT, in Fig. 5 (inset), we also present the value of *t*_*trans*_/*q*_*tetra*_ as a function of pressure. It can be found that at higher pressure above 20 kbar, *t*_*trans*_/*q*_*tetra*_ is independent of temperature and is linear with pressure. Along the isotherm of *T*_*c*_, *t*_*trans*_/*q*_*tetra*_ can be approximately expressed as a linear relationship with pressure, *t*_*trans*_/*q*_*tetra*_ = 0.0015*P* + 0.494. With decreasing pressure along the isotherm below *T*_*c*_, *t*_*trans*_/*q*_*tetra*_ departs downward from the straight line until the LLPT is encountered, while it is the opposite case above *T*_*c*_ until the liquid-vapor transition occurs.

In summary, first-principle molecular dynamics are performed for the first time to study the relationship between structural order and water-like anomalies in metastable liquid Si. Our results show that in *T*-*P* phase diagram, there indeed exists a structural anomaly region, which encloses the density anomaly but not the diffusivity anomaly. This is consistent with that of SW Si and BKS SiO_2_ but different from that of SPC/E model of water. Two-body excess entropy anomaly can neither capture the diffusivity, structural, and density anomalies, as it can in a fluid interacting through a two-scale potential. Moreover, in the structural anomaly region, *q*_*tetra*_ and *t*_*trans*_ are not perfectly correlated, which is different from that in SW Si and renders it impossible to use the isotaxis line to quantify the degree of structural order needed for water-like anomalies to occur. Along the isotherm of critical temperature *T*_*c*_, *t*_*trans*_/*q*_*tetra*_ is approximately linear with pressure, *t*_*trans*_/*q*_*tetra*_ = 0.0015*P* + 0.494. With decreasing pressure along the isotherm below *T*_*c*_, *t*_*trans*_/*q*_*tetra*_ departs downward from the line until the liquid-liquid phase transition occurs, while it is the opposite case above *T*_*c*_.

## Methods

### Ab initio molecular dynamics

The simulations are performed by the Vienna *ab initio* simulation package (VASP)^48^,^49^ together with projector augmented wave (PAW) potential^50^,^51^ in the Perdew-Burke-Ernzerhof generalized gradient approximation (PBE-GGA)^52^. A system of 216 Si atoms in a cubic supercell with periodical boundary conditions is used. The electronic wave functions are expanded in the plane wave basis set with an energy cutoff of 245 eV and a Pulay stress^53^ of 1.65 kbar is added to offset the incompleteness of the plane wave basis set. Only Γ-point is used to sample the supercell Brillouin zone. The canonical (NVT) ensemble simulations are performed with a Nosé thermostat for temperature control^54^. Newton’s equations of motion are integrated using Verlet’s algorithm in the velocity form with a time step of 2 fs.

The initial configuration, a diamond cubic crystal structure with the volume of 17.2 Å^3^/atom, is heated up to 2000 K. After a run of 20 ps with constant temperature 2000 K, the system arrives at an equilibrium liquid state. The temperature is then gradually reduced to the desired temperature with a cooling rate of 0.5 × 10^15^ K/s and a subsequent run of 15–20 ps is performed at this temperature. Then, the simulations are performed with a series of successively larger volumes along the isotherm up to the stability limit of HDL. At each simulation point, we performed a simulation about 15–30 ps. It is found that for HDL 5 ps is enough to equilibrate and the statistical averages are collected for the rest of the simulation. Along each isotherm, the end point of the previous simulation is scaled to obtain the initial conditions for the next.

### Calculation of self-diffusion coefficient D

The self-diffusion coefficient *D* in liquids was obtained by fitting the long time mean square displacement (MSD). MSD is defined as


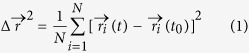


where 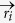 is the coordinates of atom *i, t*_0_ is an arbitrary origin of time. The diffusion coefficient *D* can be calculated by


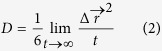


### Structural order parameters

The translational order parameter *t*_*trans*_ is defined as


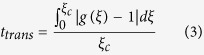


where *ξ* = *rρ*^1/3^ is the distance *r* between two Si atoms divided by the mean separation between a pair of atoms at the given number density *ρ*; *g(ξ*) is the pair correlation function, and *ξ*_*c*_ is a cutoff distance beyond which the system’s pair correlation function cannot be distinguished from its asymptotic value of 1. In this work, *ξ*_*c*_ is chosen to be 9.0 × *ρ*^1/3^. Scaled coordinates are used so that the above integral sums over an equivalent number of coordinate shells at each density. For an ideal gas, since g(r) = 1.0, *t*_*trans*_ is zero and for a crystal, it has a finite value depending on the cutoff distance. In the liquid phase, *t*_*trans*_ will have a value in between that of the ideal gas and a crystal. The tetrahedrality order parameter *q*_*tetra*_ is defined as


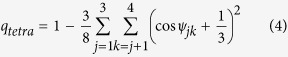


where *ψ*_*jk*_ is the angle formed by the lines joining a reference atom *i* and its nearest neighbours *j* and *k*. The average *q*_*tetra*_ varies between 0 (in the case of an ideal gas) and 1 (in the case of a cubic diamond crystal).

### Excess entropy

Excess entropy *S*_*E*_ is defined as *S*_*E*_ = *S* − *S*_*id*_, where *S* is the total entropy of the system and *S*_*id*_ is the entropy of an ideal gas system. In present work, we calculate the two body approximation (*S*_2_) to *S*_*E*_ from the pair correlation function, and is given by


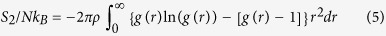


where g(r) is the pair correlation function, and *ρ* is the number density.

## Additional Information

**How to cite this article**: Zhao, G. *et al*. Relationship between structural order and water-like anomalies in metastable liquid silicon: *Ab initio* molecular dynamics. *Sci. Rep.* **7**, 39952; doi: 10.1038/srep39952 (2017).

**Publisher's note:** Springer Nature remains neutral with regard to jurisdictional claims in published maps and institutional affiliations.
